# Relict groups of spiny frogs indicate Late Paleogene-Early Neogene trans-Tibet dispersal of thermophile faunal elements

**DOI:** 10.7717/peerj.11793

**Published:** 2021-07-15

**Authors:** Sylvia Hofmann, Daniel Jablonski, Spartak N. Litvinchuk, Rafaqat Masroor, Joachim Schmidt

**Affiliations:** 1 Centre of Taxonomy and Evolutionary Research, Zoological Research Museum Alexander Koenig, Bonn, Germany; 2 Department of Zoology, Comenius University in Bratislava, Bratislava, Slovakia; 3 Institute of Cytology, Russian Academy of Sciences, St. Petersburg, Russia; 4 Zoological Sciences Division, Pakistan Museum of Natural History, Islamabad, Pakistan; 5 Institute of Biosciences, General and Systematic Zoology, University of Rostock, Rostock, Germany

**Keywords:** Phylogenetic, Paini, Himalaya, Biogeography, Paleogene, *Allopaa*, *Chrysopaa*, Tibet

## Abstract

**Background:**

The Himalaya-Tibet orogen (HTO) presents an outstanding geologically active formation that contributed to, and fostered, modern Asian biodiversity. However, our concepts of the historical biogeography of its biota are far from conclusive, as are uplift scenarios for the different parts of the HTO. Here, we revisited our previously published data set of the tribe Paini extending it with sequence data from the most western Himalayan spiny frogs *Allopaa* and *Chrysopaa* and using them as an indirect indicator for the potential paleoecological development of Tibet.

**Methods:**

We obtained sequence data of two mitochondrial loci (16S rRNA, COI) and one nuclear marker (Rag1) from *Allopaa* samples from Kashmir Himalaya as well as *Chrysopaa* sequence data from the Hindu Kush available from GenBank to complement our previous data set. A Maximum likelihood and dated Bayesian gene tree were generated based on the concatenated data set. To resolve the inconsistent placement of *Allopaa*, we performed different topology tests.

**Results:**

Consistent with previous results, the Southeast Asian genus *Quasipaa* is sister to all other spiny frogs. The results further reveal a basal placement of *Chrysopaa* relative to *Allopaa* and *Nanorana* with an estimated age of *ca.* 26 Mya. Based on the topology tests, the phylogenetic position of *Allopaa* as a sister clade to *Chaparana* seems to be most likely, resulting in a paraphyletic genus *Nanorana* and a separation from the latter clade around 20 Mya, although a basal position of *Allopaa* to the genus *Nanorana* cannot be entirely excluded. Both, the placements of *Chrysopaa* and *Allopaa* support the presence of basal Paini lineages in the far northwestern part of the HTO, which is diametrically opposite end of the HTO with respect to the ancestral area of spiny frogs in Southeast Asia. These striking distributional patterns can be most parsimoniously explained by trans-Tibet dispersal during the late Oligocene (subtropical *Chrysopaa*) respectively early Miocene (warm temperate *Allopaa*). Within spiny frogs, only members of the monophyletic *Nanorana*+*Paa* clade are adapted to the colder temperate climates, indicating that high-altitude environments did not dominate in the HTO before *ca.* 15 Mya. Our results are consistent with fossil records suggesting that large parts of Tibet were characterized by subtropical to warm temperate climates at least until the early Miocene. They contradict prevalent geological models of a highly uplifted late Paleogene proto-Plateau.

## Introduction

The uplift of the modern Himalaya-Tibet orogen (HTO) was one of the most extensive geological events during the Cenozoic. Today’s dimension of the HTO is thought to exert profound influences on the regional and global climate, and, consequently, on Asian biodiversity. Thus, understanding the evolution and knowing the past topography of the HTO is critical for exploring its paleoenvironments and historical biogeography (Kutzbach et al., 1989; Molnar, Boos & Battasti, 2010; Raymo & Ruddiman, 1992; Zhang et al., 2018). However, various lines of geoscientific evidence have suggested— partly substantially—different uplift scenarios for the respective parts of the HTO (reviewed in Spicer et al., 2020). These scenarios range from the idea of a simple monolithic rising of Tibet purely due to crustal thickening or lithosphere modification (e.g., Wang et al., 2014; Zhao & Morgan, 1985), over different models of a fractional, stepwise development (e.g., Tapponnier et al., 2001), to the concept of a high ’proto-Tibetan Plateau’ (Mulch & Chamberlain, 2006; Wang et al., 2014). Linked to these varying conceptions are uncertainties in timing, quantity (elevational increase) and sequence pattern of the HTO uplift. While several geoscientific studies present evidence for a high elevated Tibetan Plateau (TP) as early as the Eocene or even earlier (e.g., Kapp et al., 2007; Murphy et al., 1997; Tapponnier et al., 2001; Wang et al., 2008; Wang et al., 2014) others assume elevations close to modern values by the latest at the middle Oligocene (Ding et al., 2014; Quade et al., 2011; Rowley & Currie, 2006; Xu et al., 2013) or that a massive uplift occurred in the late Neogene (e.g., Molnar, England & Martiod, 1993; Su et al., 2019; Wei et al., 2016).

During the last decade, a growing number of paleontological studies provide evidence for low elevated parts of Tibet until the early Neogene or even later; for example, the presence of subtropical to warm temperate floras during the late Eocene to early Miocene have been demonstrated for the basins of Hoh Xil, Kailas, Lunpola, Nima, and Qiabulin of southern and central parts of the Plateau (Ai et al., 2019; Ding et al., 2020; Miao et al., 2016; Su et al., 2019; Sun et al., 2014; Wu et al., 2017). These findings suggest that the present high-plateau character of Tibet with its dominant alpine environments is apparently a recent formation that did not emerge before the mid-Miocene. The young ages of species divergence in the phylogenies of high-altitude taxa endemic to the plateau are a logical consequence of—and evidence for—rather recent evolution of the TP (summary in Renner, 2016; Hofmann et al., 2017; Hofmann et al., 2019). However, although it is becoming increasingly acknowledged that the HTO contributed to, and fostered, modern Asian biodiversity (Johansson et al., 2007; Steinbauer et al., 2016), our present concepts of the origin and historic biogeography of the terrestrial biotas inhabiting the HTO are far from being complete nor conclusive and have been hindered by a lack of and potential misinterpretation of data (Renner, 2016; Spicer, 2017; Spicer et al., 2020).

Phylogenies are a key mean in biogeographic and molecular evolutionary studies (Avise, 2009; Avise et al., 2000) and increasingly recognized as being essential to research that aim to reconcile biological and geological information to reconstruct Earth surface processes such as mountain building (Hoorn et al., 2013; Mulch & Chamberlain, 2018). In fact, organismal evolution offers an independent line of evidence for the emplacement of major topographical features, which have been proved valid in refining the timing of events substantiated by geologic record (Donoghue & Benton, 2007; Richardson et al., 2018). Specifically, several studies have demonstrated the suitability of phylogenetic data for addressing the timing and complexity of orogenic events, e.g., the Andean uplift and the formation of the Qinghai-Tibetan region (Antonelli et al., 2009; Luebert & Muller, 2015).

We here use spiny frogs of the tribe Paini (Dicroglossidae) to untangle the spatiotemporal evolution of this group in the HTO and, thus, as an indirect indicator for the topographic and paleoecological development of High Asia. Spiny frogs occur across the Himalayan mountain arc from northern Afghanistan, Pakistan, and northern India, through Nepal, Sikkim, and Bhutan, and in the valleys of southern and eastern Tibet, eastwards to eastern China, and southwards to the mountains of Indochina (Myanmar, Thailand, Laos, northern Vietnam; Frost, 2021). They live mostly in boulder-rich running water (Dubois, 1975) or clear pools with flowing water. Males are characterized by black, keratinous spines (Ohler & Dubois, 2006). The Paini tribe is currently composed of the genus *Nanorana* Günther, 1896 (around 30 species), *Quasipaa* Dubois, 1992 (11 species), *Allopaa* (Ohler & Dubois, 2006) (possibly two species), and the monotypic genus *Chrysopaa* (Ohler & Dubois, 2006). Following Che et al. (2010) and our own findings (Hofmann et al., 2019), *Nanorana* can be subdivided into three subgenera (*Nanorana*, *Paa*, and *Chaparana*). However, the phylogenetic and mostly taxonomic relationships among Paini are not completely resolved with several taxonomic changes during the last decade including taxa descriptions (Che et al., 2009; Frost, 2021; Huang et al., 2016; Jiang et al., 2005; Pyron & Wiens, 2011).

Previous studies proposed contrasting hypotheses to explain the current distributional and phylogenetic patterns of spiny frogs in the HTO. While a strict vicariance driven scenario suggests species formation among major lineages when the species were “trapped” in the mountain mass and become separated when it uplifted (Che et al., 2010), a more recent study found no clear support for this model but indications for a Paleo-Tibetan origin of Himalayan spiny frogs (Hofmann et al., 2019), confirming modern hypotheses for the past topographic surfaces of the southern parts of the HTO. This Tibetan-origin scenario (Schmidt et al., 2012) assumes that adaptation of Himalayan spiny frogs to the high-altitude environment occurred in South Tibet, at a time when the Greater Himalaya had not yet risen to its present height (Hofmann et al., 2019). With the continuously uplifting Himalaya along with the drying of southern Tibet, these ancestral lineages have probably been forced to follow the spatially shifted suitable habitats along the transverse river valleys of the Himalayas, such as the Brahmaputra, Kali Gandaki, or the Indus catchment (Hofmann et al., 2019). The hypothesis about the South-Tibetan origin has been also demonstrated in other Himalayan faunal elements, e.g., *Scutiger* lazy toads (Hofmann et al., 2017) and forest-dwelling *Pterostichus* ground beetles (Schmidt et al., 2012).

So far, the phylogenetic placement of the westernmost Dicroglossid frogs that occur in the HTO (*Allopaa* from Kashmir Himalaya and *Chrysopaa* from Hindu Kush) has never been addressed. Given the Tibetan-origin hypothesis (Hofmann et al., 2017; Schmidt et al., 2012), we expect that thermophile clades from the northwestern margin of the HTO represent distinct lineages and are not closely related to any of the geographically neighbouring lineages that occur in the Himalaya and on the central Tibetan Plateau. If so, a basal placement of these westernmost groups relative to other Himalayan Paini or a close relationship to thermophile taxa from the east of the Himalayan arc would argue for Paleocene dispersal of warm temperate or subtropical lineages westward to the northwestern margin of the HTO. Thus, integrating *Allopaa* and *Chrysopaa* into the analysis would allow better understanding of the time at which spiny frogs have adapted to high mountains and about which part of the paleo-HTO was occupied first by these amphibians. Consequently, the phylogeny of these frogs is of particular interest with respect to the controversial debate regarding the geological and paleoecological development of High Asia (see above). Therefore, we here reanalysed our previous dataset (Hofmann et al., 2019) by extending it with sequence data from *Allopaa* and *Chrysopaa*. We use our findings of the Paini phylogeny and time tree to discuss the biogeographic history of these frogs against the background of current HTO uplift concepts.

## Materials & Methods

### Sampling, laboratory protocols and data acquisition

**Figure 1:**
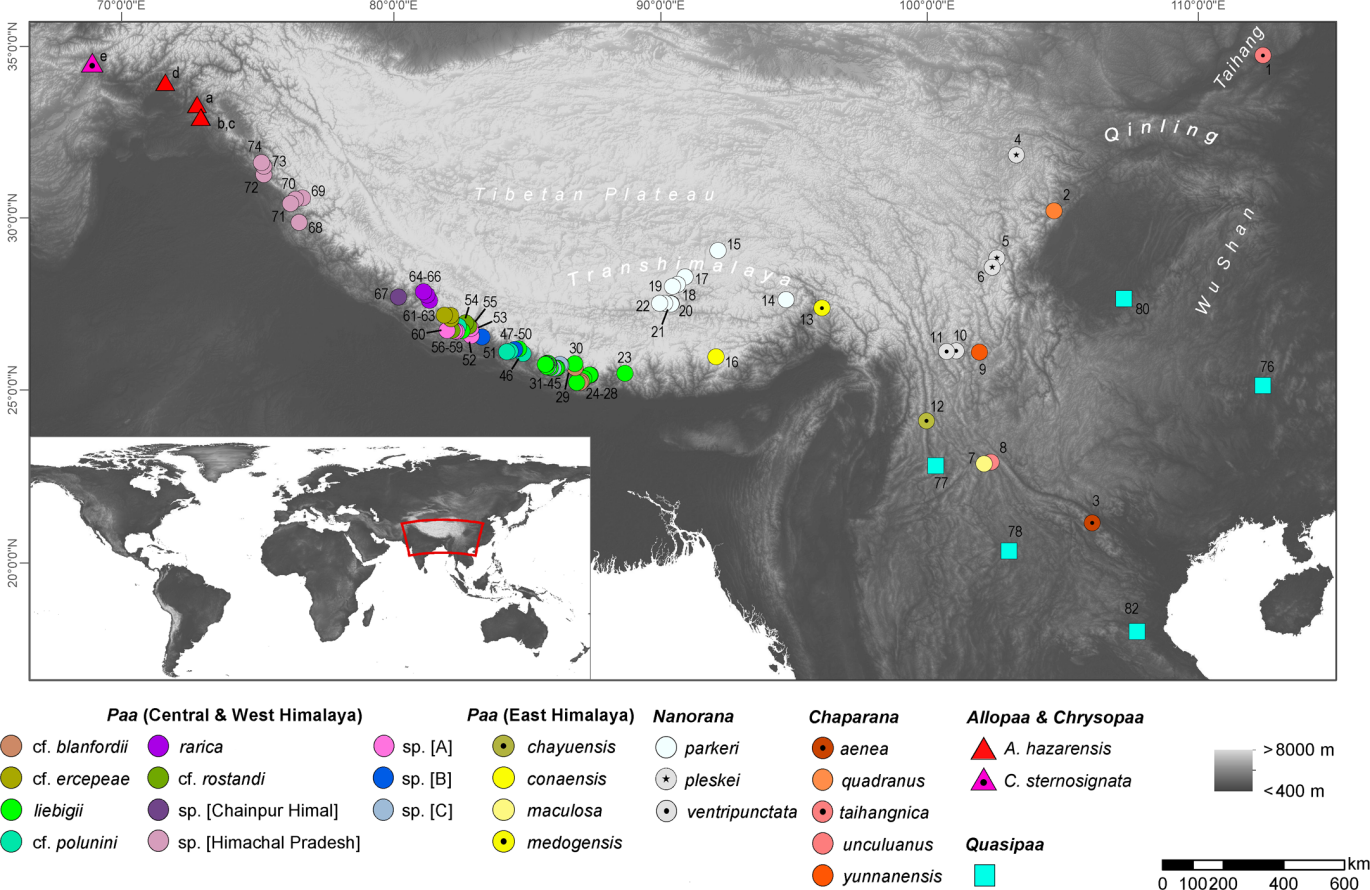
Map showing the origin of sequence data used in this study; locality numbers refer to samples and sequences listed in Table S1.

We used sequence data of the 16S ribosomal RNA (rRNA), mitochondrial Cytochrome c oxidase I (COI) and nuclear Recombination activating gene 1 (Rag1) region available from our previous study (Hofmann et al., 2019) and complemented the data with a newly generated sequences for these three gene regions from *Allopaa hazarensis* (Dubois, 1975) (*n* = 6; Pakistan, including the type locality of the species - Datta, Manshera District, Hazera Division; for details see Fig. 1 and Table S1). Sampling was performed under the permit of the Pakistan Museum of Natural History, Islamabad, Pakistan (No. PMNH/EST-1(89)/05), according to the regulations for the protection of terrestrial wild animals. We also included 16S rRNA and COI sequence data of *Chrysopaa sternosignata* from Bagram, Parwan Province, Afghanistan (Hindu Kush Mts.) available in NCBI GenBank (accession numbers: MG700155 and MG699938). Our *Nanorana* samples from Himachal Pradesh, which were previously referred to as “sp.” (Hofmann et al., 2019), were identified as *Nanorana vicina* based on morphological characters (Boulenger, 1920; Stoliczka, 1872); for photos of live specimens Fig. S1. Genomic DNA was isolated from ethanol tissues using the DNeasy Blood & Tissue Kit (Qiagen, Venlo, Netherlands) according to the manufacturer’s protocol. Approximately 571 bp of the 16S, 539 bp of the COI, and a sequence segment of 1,207 bp of Rag1 gene were amplified using primers and PCR conditions as previously described (Hofmann et al., 2019). Amplicons were purified using the ExoSAP-IT enzymatic clean-up (USB Europe GmbH, Staufen, Germany) and the mi-PCR Purification Kit (Metabion, Planegg, Germany) or directly purified by Eurofins Genomics (Germany) with in-house protocols. The Sanger sequencing was performed on an ABI 3730 XL sequencer at Eurofins Genomics or by Macrogen Inc. (Seoul, South Korea or Amsterdam, The Netherlands; http://www.macrogen.com).

### Sequence alignment and phylogenetic reconstruction

We aligned our new 16S sequences to the previous secondary structures-based data set (Hofmann et al., 2019) by eye; sequences of the protein-coding genes were aligned using the MUSCLE algorithm (Edgar, 2004) in MEGA X (Kumar et al., 2018). No ambiguities, such as deletions, insertions, or stop codons, were found neither in the alignment based on nucleotides nor in amino acids alignment.

The concatenated rRNA + mtDNA + nuDNA sequence alignment consisted of 184 taxa and contained 2,317 nucleotide positions of which 494 were phylogenetically informative. Nuclear data were unphased as most of the taxa were represented by only single individuals. We inferred a maximum-likelihood (ML) and a Bayesian inference (BI) tree based on the concatenated sequence data using RAxML v.8.2.12 (Stamatakis, 2014), IQ-TREE v.2.0 and MrBayes v.3.2.6 (Ronquist et al., 2012). We partitioned the dataset a priori by gene and codon fragments and used PartitionFinder 1.1.1 (Lanfear et al., 2012) to optimize the partition scheme with the following setting: branch lengths linked, corrected Aikaike Information Criterion (AICc), greedy search algorithm, and the substitution models implemented in RAxML and MrBayes. RAxML was run with the GTRGAMMA model and 1,000 bootstrap replicates on the CIPRES (Cyberinfrastructure for Phylogenetic Research) (Miller, Pfeiffer & Schwartz, 2010). IQ-TREE was performed with the edge-linked partition model (Chernomor, von Haeseler & Minh, 2016) and both SH-like approximate likelihood ratio test (SH-aLRT) (Guindon et al., 2010) and the ultrafast bootstrap approximation (Hoang et al., 2018) using 1 Mio replicates per test. In the Bayesian analysis we assigned the doublet model (16 ×16) proposed by Schoniger & von Haeseler (1999) to the rRNA stem regions. Unambiguous stem pairs were inferred based on the consensus structure from RNAsalsa 0.8.1 (Stocsits et al., 2009) and implemented in the MrBayes input file. For the analysis of the remaining positions, the standard 4 ×4 option was applied using a GTR evolutionary model for all nucleotide partitions. The site-specific rates were set variable. For reasons of comparison, we also inferred the Bayesian tree using the 4 ×4 standard model of DNA substitution for all regions and the optimized models and partitions as suggested by PartitionFinder. MrBayes was run with a random starting tree for five million generations, sampling trees every 500th generation. Inspection of the standard deviation of split frequencies as well as an effective sample size value >200 of the traces using Tracer v. 1.7.1 (Rambaut et al., 2018) indicated convergence of Markov chains. In all analyses, we used four parallel Markov chain Monte Carlo simulations with four chains (three heated and one cold) and discarded the first 25% of the samples of each run as burn-in; consensus trees were produced using the sumt command.

To test competing topologies, we used a Bayes Factor (BF) approach and the tree topology tests implemented in IQ-TREE, namely the approximately unbiased (AU) test (Shimodaira, 2002) as well as the RELL approximation (Kishino, Miyata & Hasegawa, 1990), including bootstrap proportion, Kishino-Hasegawa test (Kishino & Hasegawa, 1989), Shimodaira-Hasegawa test (Shimodaira & Hasegawa, 1999), and expected likelihood weights (Strimmer & Rambaut, 2002). The marginal likelihoods estimations (MLE) for the BF calculations were obtained under each model based on both the stepping-stone (ss Xie et al., 2011) and path sampling (ps Lartillot & Philippe, 2006) methods implemented in BEAST v.1.10.4 (Suchard et al., 2018) using optimal partitions and substitution models as assessed in PartitionFinder, 250 million generations, a logging interval of 25,000, a MLE chain length of 1 million, and 100 path steps. Statistical support was then evaluated via 2lnBF using the ps/ss results as per Kass & Raftery (1995). Finally, we also used the stepping-stone approach with 10 million generations (4 runs and 4 chains), to estimate the model likelihood values for BF calculation with MrBayes by implementing the doublet option on 16S rRNA stem regions and the standard substitution option on all other regions. We specifically tested the hard constraint *vs.* negative constraint on *Chaparana* and *Allopaa*. In statistical hypothesis testing, models are compared to assess the strength of evidence against the null hypothesis (H_0_), which is defined as the one with the lower marginal likelihood (i.e., with the smaller value of the negative log-likelihood): 2lnBF <2 implies no evidence against H_0_; 2–6, weak evidence; 6–10, strong evidence; and >10 very strong evidence. For the RELL approximation we used 1 Mio replicates, all other settings were left as default.

### Molecular dating

Divergence dates were estimated using BEAST2 v.2.6.2 (Bouckaert et al., 2014), based on the full concatenated dataset because of missing data in the alignment for some of the taxa (see Hofmann et al., 2019). Similar as to the MrBayes analyses, the partition scheme was optimized using PartitionFinder and the models that are implemented in BEAST. It is not possible to consider secondary structure information in BEAST (ambiguities are treated as unknown data so we did not remove stem regions)—thus all positions of the respective rRNA partition were treated under the same evolutionary model. Age constraints were derived from our previous calibration analysis of the phylogeny of *Nanorana*, which based on fossil-calibrated divergence estimates (Hofmann et al., 2019): MRCA of Paini 38.10 Ma, 28.70–47.50 (normal, sigma: 4.80); split of Tibetan *Nanorana* and Himalayan *Paa* 12.59 Ma, 7.93–17.30 (normal, sigma: 2.38); separation of the Plateau frog (*N. parkeri*) and *N. ventripunctata*+*N. pleskei* 6.35 Ma, 3.54–9.16 (normal, sigma: 1.44).

Analysis relied on ten independent BEAST runs with a chain length of 50 million, a thinning range of 5,000, a lognormal relaxed clock model, a Yule tree prior, a random starting tree, and the site models selected by bModelTest package (Bouckaert & Drummond, 2017) implemented in BEAST2. Runs were then combined with BEAST2 LogCombiner v.2.6.2 by resampling trees from the posterior distributions at a lower frequency, resulting in 9,010 trees. Stationary levels and convergence of the runs were verified with Tracer based on the average standard deviation of split frequencies and ESS values >200. The final tree was obtained with TreeAnnotator v.2.6.2 and visualized with FigTree v.1.4.3 (Drummond & Rambaut, 2007).

## Results

### Phylogeny of Paini from the HTO

**Figure 2:**
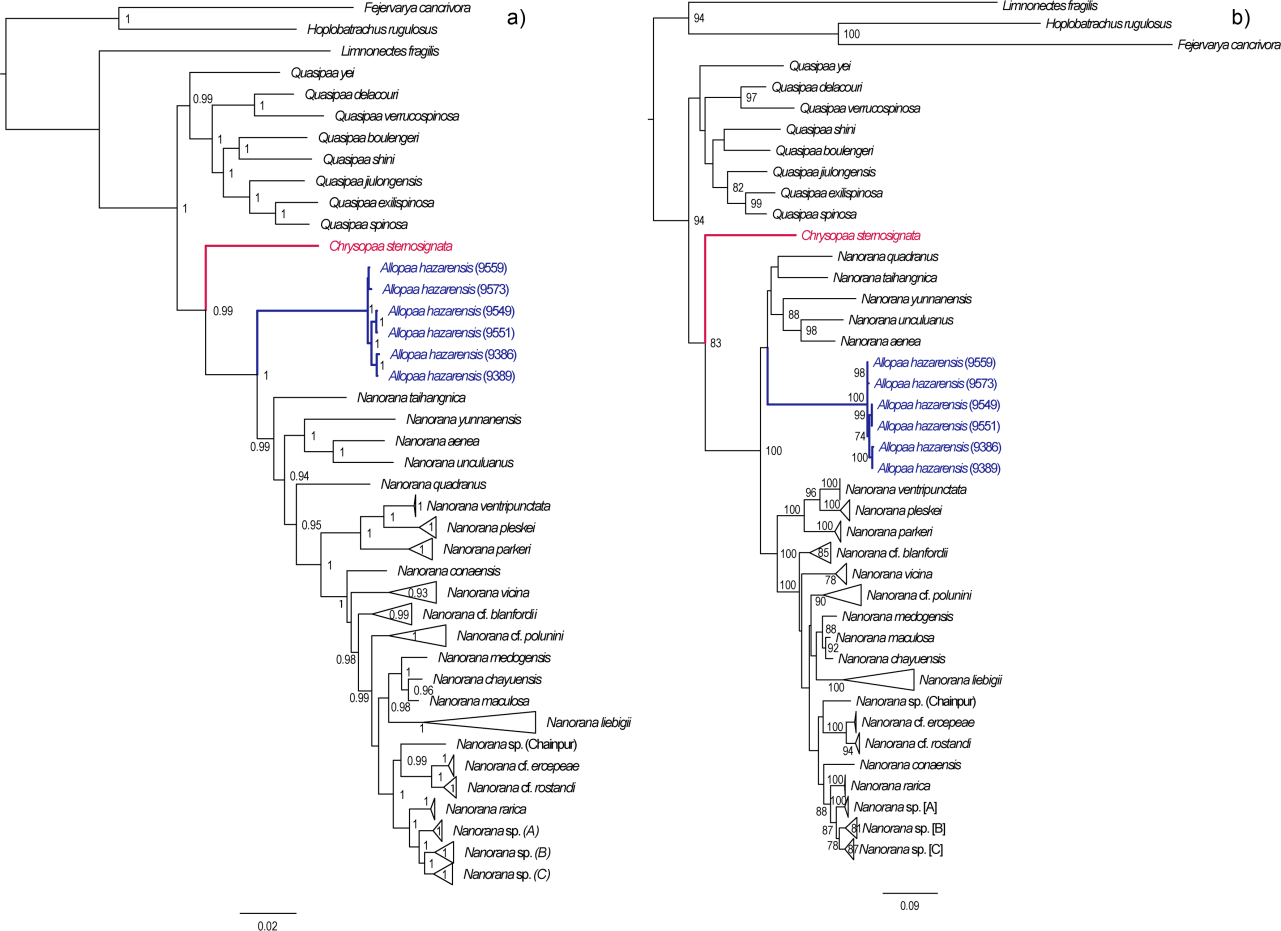
Bayesian inference (A) and Maximum-likelihood (B) tree inferred from the concatenated mtDNA and nuDNA sequence alignment. Numbers at branch nodes refer to posterior probabilities ≥ 0. 9 and bootstrap values > 70, respectively. For IQ-TREE topology see supplemental Fig. S2.

In both the ML and BI analyses, a relatively well resolved tree was obtained with strong support for most of the main clades, although with partly inconsistent and uncertain branching patterns of lineages within (sub)clades (Fig. 2). When information on secondary structure of 16S rRNA is considered (BI-tree), the results strongly support three monophyletic clades within Paini, apart from the monotypic *Chrysopaa*: *Quasipaa*, *Allopaa*, and *Nanorana*, with *Allopaa* forming the sister taxon to all *Nanorana*. Otherwise, *Allopaa* clusters with *Chaparana*, which together form the sister clade to *Paa* and *Nanorana* subgenera in the ML-tree (see also Fig. S2 for topology generated with IQ-TREE and with MrBayes using the 4 ×4 substitution model, and Fig. S3 for ML trees based on 16S+COI and on Rag1 sequence data). The most striking result, consistently recovered in all trees, is the placement of *Chrysopaa* from the northern-central Afghanistan (Hindu Kush Mts.), which forms the sister taxon to *Allopaa* and *Nanorana*.

In accordance with our previous findings, three monophyletic subclades can be distinguished within *Nanorana*, namely *Chaparana* from montane regions of the southeastern margin of the TP and mountains of NE China, *Paa* from high-montane regions of the West, Central and East Himalaya*,* and nominal *Nanorana* from subalpine and alpine regions of the TP and its eastern margin. Monophyly of *Chaparana* is not supported in the analyses if secondary structure of 16S is ignored. All *Paa* species together form the most species diverse clade.

Since the placement of *Allopaa* is of particular interest in terms of the origin and past biogeography of Paini, we tested the resulting topologies of major clades: BI tree considering secondary structure information of 16S, t_1_: (*Allopaa* (*Nanorana*)); RAxML/BI without secondary structure information, t_2_: ((*Chaparana*, *Allopaa*)(*Nanorana* sensu stricto, *Paa* sensu stricto)).

The AU test does not reject one of the two placement models for *Allopaa* (Table 1), as do the results of all other IQ-TREE tests. However, the BF of 28 (ss) and 32 (ps), based on the model likelihood values estimated with BEAST, strongly rejects a basal placement of *Allopaa* relative to the genus *Nanorana* in favor of the topology seen in the ML tree. Similarly, the marginal likelihoods calculated based on the runs considering the secondary structure of 16S were significantly higher for the unconstraint model (Table 1). Thus, the phylogenetic position of *Allopaa* as sister clade to *Chaparana* seems to be most likely, thereby making the *Nanorana* genus paraphyletic.

**Table 1:** Tree topology comparisons between the two models of *Allopaa* placements. Models (*t*_1_, *t*_2_) are compared based on Bayesian factor (BF) using BEAST, as well as the unbiased (AU) test (Shimodaira 2002), bootstrap proportion using RELL method (Kishino, Miyata & Hasegawa, 1990), Kishino-Hasegawa (KH) test (Kishino & Hasegawa, 1989), Shimodaira-Hasegawa (SH) test (Shimodaira & Hasegawa, 1999), and expected likelihood weights (ELW) using IQ-TREE; BF was also calculated for a hard constraint on *Chaparana* and *Allopaa* (A+Ch) *vs*. an unconstraint constellation using the stepping-stone approach in MrBayes and considering the secondary structure information of 16S.

Topology	ps	ss	2lnBF	logL	deltaL	bp-RELL	p-KH	p-SH	c-ELW	p-AU
t_1_ (A(N))	−15471	−15477	ps: 32	−14164.109	2.458	0.397+	0.383+	0.383+	0.399+	0.383+
t_2_ ((A+Ch)(P,N))	**−15455**	**−15463**	ss: 28	**−14161.652**	0	0.603+	0.617+	1+	0.601+	0.617+
(A+Ch)		**−16472**	ss: 56							
unconstraint		−16500								

**Notes.**

A *Allopaa* C *Chaparana* N *Nanorana* (genus) P *Paa* ps path sampling log marginal likelihood ss stepping-stone log marginal likelihood + a tree is not rejected if its *p*-value > 0.05

Bold log marginal likelihood values indicate the model most favored by a method (higher is better).

### Divergence times in spiny frogs

**Figure 3:**
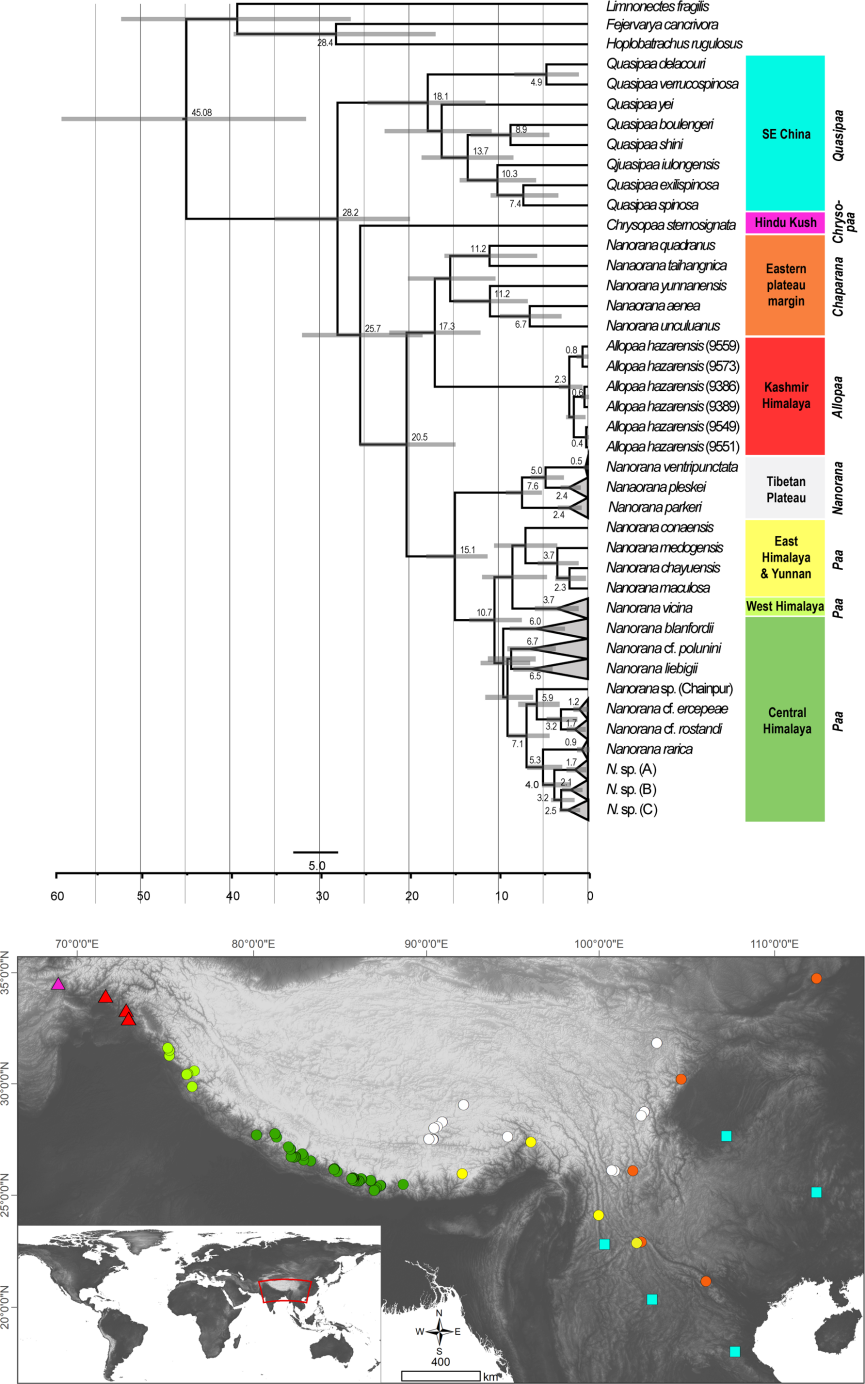
Ultametric time-calibrated phylogeny generated with BEAST2 based on the concatenated sequence data of spiny frogs. Grey bars specify the 95% HPD for the respective nodes; ages are shown for nodes that are supported by Bayesian posterior probability ≥0.95. Colors at clades correspond to the lower distribution map.

Dating analysis suggests an origin of Paini (*Allopaa, Chrysopaa, Nanorana, Quasipaa*) in the mid Oligocene (28.21 Ma, 20.11–35.18 Ma), what is in the range of previous estimations (Che et al., 2010; Hofmann et al., 2019; Sun et al., 2018) (Fig. 3). The age of Himalayan-Tibetan spiny frogs (*Allopaa, Chrysopaa, Nanorana*) is estimated to be 25.7 Ma (18.70–32.16). Within crown *Allopaa*+*Nanorana*, the clade comprising the montane *Chaparana* and West-Himalayan *Allopaa* split from the Central/East Himalayan and Tibetan *Nanorana* (subgenera *Paa* and *Nanorana*) in the early Miocene, around 20 Ma, followed by the separation of *Chaparana* and *Allopaa ca.* 3 million years later. The divergence of the nominal *Nanorana* (endemic to the TP) from *Paa* (Greater Himalaya) occurred around 15 Ma (11.45–18.27 Ma). This estimate is close to the age of 13 Ma (7–25 Ma) calculated by Sun et al. (2018), and 10–12 Ma estimated by Wiens et al. (2009).

Diversification of Central Himalayan *Paa* clades has taken place continuously during the whole Mid to Late Miocene. Most of the main lineages within *Paa* were present at least in the late Miocene, and nearly all species are not younger than the Pliocene.

## Discussion

We here report the first phylogeny of the westernmost HTO Paini taxa *Chrysopaa sternosignata* and *Allopaa hazarensis* in the context of their closest relatives. Our work based on sequence information of *A. hazarensis* specimens from the foothills of the Kashmir Himalaya, a previously published data set (Hofmann et al., 2019), and additional sequence data of *C. sternosignata* from the Hindu Kush Mts. in Afghanistan available from GenBank. The study provides evidence for an early-Miocene evolution of Himalayan Paini, which is ultimately linked to the paleoecological evolution of the HTO.

Consistent with our previous results (Hofmann et al., 2019), the Southeast Asian genus *Quasipaa* is sister to all other spiny frogs. Most remarkable, the monotypic *Chrysopaa* is placed basally relative to *Nanorana* and *Allopaa*, supporting the presence of ancestral Paini lineages in the far northwestern part of the HTO, which is diametrically opposite end of the HTO with respect to the ancestral area of spiny frogs that is assumed to be the Paleogene East or Southeast China (Che et al., 2010; Hofmann et al., 2019). Thus, it can be assumed that the ancestor of *Chrysopaa* appeared elsewhere near the eastern margin of the HTO during the late Oligocene-early Miocene. If so, it implies that members of the *Chrysopaa* stem group must have been temporarily present in the interior of the HTO during the following time, to enable a range expansion up to the western margin of the mountains system. Given this scenario, the climatic preferences of ancestral spiny frogs are of particular interest. Amphibians are particularly sensitive to changes in hydric and thermal environmental conditions (Kerby et al., 2010; Mitchell & Janzen, 2010; Ochoa-Ochoa et al., 2012; Stuart et al., 2004), and many of them show remarkable evolutionary stasis in ecological niches, suggesting that dispersal might have been historically constrained between similar climatic conditions (Konzak & Wiens, 2010; Wiens, 2011, and references therein). Since all species of the most basal clade *Quasipaa* occur under subtropical climate (Frost, 2021; Bain & Hurley, 2011), a similar temperature preference might be assumed for the *Chrysopaa* ancestor. We suspect that this preference has not changed significantly during the Neogene period as *C. sternosignata* occurs under subtropical to warm temperate climate conditions in the colline zone of the Hindu Kush Mts. and the Kashmir valley (Khan, 2006; Sarwar et al., 2016; Wagner et al., 2016). Consequently, a subtropical climate associated with sufficient humidity suitable for amphibians might have existed in large parts of the late Oligocene-Tibet to allow a trans-Tibetan dispersal of *Chrysopaa* stem group members. Interestingly, basal divergences of West Himalayan taxa are also known from the gekkonid genus *Cyrtodactylus*, dating even back to the early Eocene and demonstrating that ancestral *Cyrtodactylus* were present in the “proto-Himalayan region” (Argawal et al., 2014). The topology of the genus provides striking parallels to the Paini tree and indications in support of a Tibetan-origin (Hofmann et al., 2017; Schmidt et al., 2012; Hofmann et al., 2019) of *Cyrtodactylus* followed by a trans-Tibetan dispersal of ancestral lineages to the northwestern HTO margin.

Also unexpected are our results with respect to the phylogenetic position and timing of the evolution of *Allopaa* from the foothills of the Kashmir Himalaya. This group evolved during the early to mid-Miocene most parsimoniously as sister clade to *Chaparana*, although a basal position of *Allopaa* relative to the genus *Nanorana* cannot be entirely excluded. Species of *Chaparana* occur along the eastern margin of the HTO and therewith at the opposite end of the HTO where *Allopaa* is distributed. A similar paradoxical pattern can be found in the above mentioned *Cyrtodactylus* group (Argawal et al., 2014) and in Broscini ground beetles (Schmidt, Wrase & Sciaky, 2013) with species from the western Himalaya being most closely related to those from the Eastern Himalaya and Southeast Asia. *Chaparana* and *Allopaa* together constitute most likely the sister clade to the Tibetan *Nanorana* and Himalayan *Paa*, which indicates that *Nanorana* might be paraphyletic with respect to *Allopaa.* However, to prevent instability in taxonomic nomenclature, at this stage we refrain from proposing any taxonomic changes until further evidence is available. Our results also show that *Allopaa* is phylogenetically not related to the biogeographically neighboring Himalayan spiny frogs. This finding is crucial with respect to the ancestral distributional area of the *Chaparana*+*Allopaa* clade and their ancestral habitat preferences. Recent species of *Chaparana* occur in the colline and lower montane zone along the eastern margin of the HTO and the easterly neighbored mountains and, thus, immediately adjacent to (or overlapping with) the supposed ancestral area of spiny frogs (Che et al., 2010; Hofmann et al., 2019). Similar as assumed for *Chrysopaa*, the ancestor of *Allopaa* must have been dispersed across a moderately elevated Tibetan Plateau, although about eight million years later than the ancestor of *Chrysopaa*. Since species of *Allopaa* occur under warm-temperate conditions in the colline to lower montane zone (comparable to those of its sister group *Chaparana*; Ahmed et al., 2020), similar temperature preferences can be assumed for ancestral *Allopaa*. Therefore, the supposed trans-Tibet dispersal event of this lineage implies the presence of warm temperate conditions in significant parts of Tibet’s interior at least up to the early-mid Miocene boundary. This is supported by the evidence of subtropical to warm-temperate fossil floras in the Qiabulin basin at 21–19 Ma (Ding et al., 2014), Lunpola basin at 25.5–19.8 Ma (Sun et al., 2014) and in the Kailas basin at about 23.3 Ma (Ai et al., 2019). Due to the progressive uplift of Tibet and the associated continuous cooling of the regional climates, the *Allopaa* stem group members might have successively been lost to extinction. Today’s absence of members of *Chaparana* and *Allopaa* in the high montane zone throughout the HTO suggests that these lineages were probably not able to adapt fast enough to the conditions that resulted from the dramatically changing environment. Alternatively, a westward and northwestward spread of ancestral *Allopaa* using subtropical to warm-temperate habitats which paralleling the southern slopes of the Himalaya must also be considered. However, this model is very unlikely, as it would imply extinction of all ancestral lineages in fast areas covering almost the whole Himalayan mountain arc. Considering that since the onset of surface uplift subtropical to warm temperate environments were continuously present along the Himalayan southern face (Hongfu, 1994; Sanyal & Sinha, 2010; Xu et al., 2012), such radical extinction or turnover is implausible given the recent and former ecological conditions in this area. Moreover, the absence of *Allopaa*, but occurrence of many spiny frogs of the subgenus *Paa* along the southern slopes of the eastern, central, and western Himalaya north to the Indian Himachal Pradesh, contradicts this extinction scenario.

Unlike spiny frogs of the taxa *Chrysopaa*, *Allopaa* and *Chaparana* which are restricted to the subtropical to warm temperate climate, many representatives of the *Nanorana* +*Paa* clade are adapted to colder habitats and occur in the high montane, subalpine, and alpine zones of the HTO. The evolutionary late appearance of this clade is indicative for the minimum age of high-altitude environments in the HTO: Although spiny frogs were present in the area since at least the Paleogene/Neogene boundary, cold-adapted species did not evolve before *ca*. 15 Ma (Fig. 3). This is a strong hint that extensive high-altitude environments were present in the HTO from mid-Miocene at earliest.

## Conclusions

We provide the first phylogenetic study of spiny frogs that comprises the two westernmost Himalayan taxa *Allopaa* and *Chrysopaa*. Our findings suggest a late Oligocene to early Miocene dispersal of two subtropical respective warm temperate lineages, *Chrysopaa* and *Allopaa*, from the ancestral area of spiny frogs in SE Asia across the HTO into its far northwestern part. This dispersal scenario is crucial with respect to the long-standing debate regarding the paleoenvironmental and paleoelevational development of the TP. Given the stem age of subtropical *Chrysopaa* of *ca*. 26 Mya and the divergence time of 17 Mya between warm temperate *Allopaa* and *Chaparana*, our results strongly indicate the large-scale presence of subtropical environments north of today’s Greater Himalaya until the late Oligocene, and of warm temperate climates until the late Miocene. This contrasts with geoscientific models of the paleoelevational evolution of the TP which assume large scale surface uplift close to present heights until the mid-Oligocene (e.g., Kapp et al., 2007; Mulch & Chamberlain, 2006; Tapponnier et al., 2001; Wang et al., 2008; Wang et al., 2014), and which are widely used in recent biogeographic studies to develope evolutionary scenarios in different species groups (e.g., Favre et al., 2015; Favre et al., 2016; Renner, 2016; Mosbrugger et al., 2018). However, over the last decade a growing number of fossil data provide evidence for the presence of tropical to warm temperate floras and freshwater fishes in central Tibet during the late Paleogene until the early Neogene (Song et al., 2010; Su et al., 2019; Wei et al., 2016; Wu et al., 2017). Consistent with these findings our results support the recent concept proposed by Spicer and colleagues (Spicer et al., 2020), which assumes that the TP was not uplifted as a whole, but instead, a deep wide east–west oriented valley occurred in the Tibetan interior before the final plateau formation. We suspect that this supposed valley represents the migration corridor of the ancestral *Chrysopaa* and *Allopaa* lineages, which today are represented by the two relict taxa, *C. sternosignata* and *A. hazarensis*, endemic to the region of the Hindu Kush and Kashmir Himalaya. This scenario is in line with and adds to the Tibetan-origin hypothesis of the paleo-Tibetan fauna (Hofmann et al., 2017; Hofmann et al., 2019; Schmidt, Wrase & Sciaky, 2013). Disjunct distribution patterns of species groups between the eastern and western part of the HTO, as we demonstrate here for spiny frogs, have been also observed in *Cyrtodactylus* (Argawal et al., 2014, see Discussion), and in Broscini ground beetles, with the genus *Eobroscus* widely distributed in East Asia and Indochina and with *Kashmirobroscus* endemic to a small part of the Kashmir Himalaya (Schmidt, Wrase & Sciaky, 2013). Moreover, the Kashmir Himalaya is well-known for the occurrence of several highly endemic ground beetles (Schmidt et al., 2012). We expect that numerous additional lineages endemic to the Kashmir Himalaya will be identified in future which may contribute to resolve the evolution of the HTO. We therefore encourage further and systematic research in this area and the use of more powerful molecular data, for example, through the use of genomic sequencing to better understand the evolution and Cenozoic history of Himalayan biodiversity against the background of existing geological scenarios.

##  Supplemental Information

10.7717/peerj.11793/supp-1 Supplemental Information 1 Photo vouchers of *Allopaa hazarensis* (left panels) and *Nanorana vicina* (right panels) from the present study Sampled for DNA in Pakistan and Himachal Pradesh, respectively (Photographs of *Allopaa hazarensis*: D. Jablonski; of *Nanorana vicina*: S. Litvinchuk): (A) *A. hazarensis* from the type locality Datta, Pakistan (1,300 m; locality no. a); (B) *A*. *hazarensis* from Margi, Murree, Pakistan (1618 m; locality no. c); (C) *A*. *hazarensis* from locality Laram Qilla, Lower Dir, Pakistan (1,436 m; locality no. d); (D) tadpole of *A. hazarensis* from Margi, Murre, Pakistan (1,618 m, locality no. c); E) *N. vicina* from Narkanda (2,650 m; ,locality no. 68); (F) *N. vicina* from Pulga, (2199 m; locality no. 69); (G) *N. vicina* from Panjpula (2,016 m; locality no. 72); (H) tadpole of *N. vicina* from Panjpula (2,016 m; locality no. 72).

10.7717/peerj.11793/supp-2 Supplemental Information 2 IQ TREE topology (A); MrBayes tree generated with the standard 4 ×4 model of DNA substitution (B)

10.7717/peerj.11793/supp-3 Supplemental Information 3 ML tree topology

10.7717/peerj.11793/supp-4 Supplemental Information 4 List of species used in the present study, including sample ID or voucher numbers, sample localities and GenBank accession numbers Locality identifier (Loc) refer to the Fig. 1. Coordinates are given in decimal degrees. §= samples of photographed specimens (see Fig. S1); * = sample localities not shown in Fig. 1 because they lie far to SO China; for reference see map in Hofmann et al. (2019).

## Additional Information and Declarations

### DNA Deposition

The following information was supplied regarding the deposition of DNA sequences: The data are available at GenBank (Table S1).

### Data Availability

The following information was supplied regarding data availability: The data are available at GenBank (Table S1). The analysis pipelines used in this study are publicly accessible:
https://www.megasoftware.net/;
https://cme.h-its.org/exelixis/web/software/raxml/;
http://www.iqtree.org/;
https://nbisweden.github.io/MrBayes/download.html;
https://www.robertlanfear.com/partitionfinder/;
https://beast.community/;
https://www.beast2.org/.
