# Supplementary material for: Relict groups of spiny frogs indicate Late Paleogene-Early Neogene trans-Tibet dispersal of thermophile faunal elements
Source: PeerJ. 2021 Jul 15;9:e11793. doi: 10.7717/peerj.11793 (PMC8286701; doi:10.7717/peerj.11793)
Supplement: Supplemental Information 1 — Sampled for DNA in Pakistan and Himachal Pradesh, respectively (Photographs of Allopaa hazarensis: D. Jablonski; of Nanorana vicina: S. Litvinchuk): (A) A. hazarensis from the type locality Datta, Pakistan (1,300 m; locality no. a); (B) A. hazarensis from Margi, Murree, Pakistan (1618 m; locality no. c); (C) A. hazarensis from locality Laram Qilla, Lower Dir, Pakistan (1,436 m; locality no. d); (D) tadpole of A. hazarensis from Margi, Murre, Pakistan (1,618 m, locality no. c); E) N. vicina from Narkanda (2,650 m; ,locality no. 68); (F) N. vicina from Pulga, (2199 m; locality no. 69); (G) N. vicina from Panjpula (2,016 m; locality no. 72); (H) tadpole of N. vicina from Panjpula (2,016 m; locality no. 72). [file peerj-09-11793-s001.pdf]

Supplemental Information Figure S1

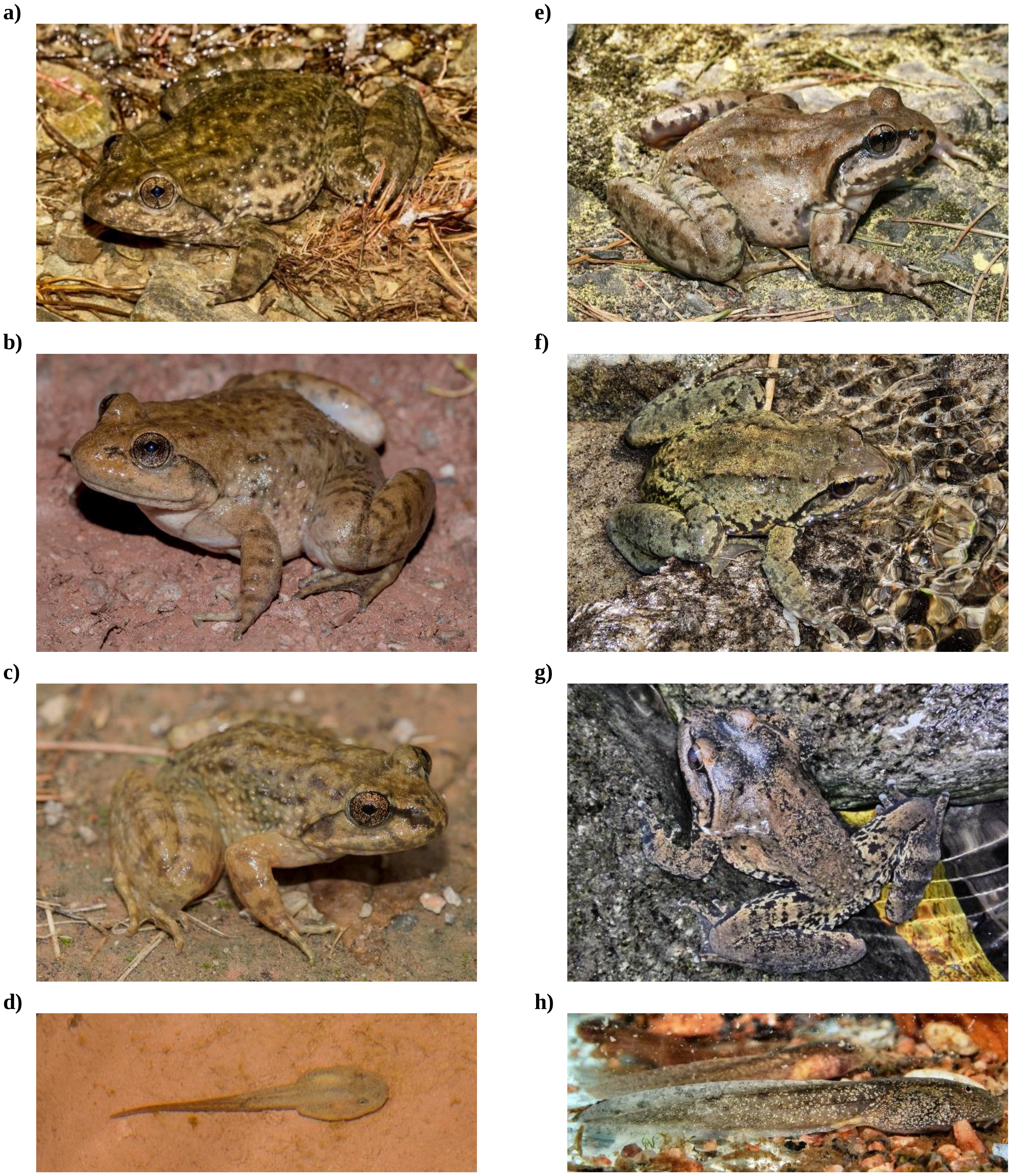

Photo vouchers of *Allopaa hazarensis* (left panels) and *Nanorana vicina* (right panels) from the present study, sampled for DNA in Pakistan and Himachal Pradesh, respectively (Photographs of *Allopaa hazarensis*: D. Jablonski; of *Nanorana vicina*: S. Litvinchuk): **a)** *A. hazarensis* from the type locality Datta, Pakistan (1300 m; locality no. a); **b)** *A. hazarensis* from Margi, Murree, Pakistan (1618 m; locality no. c); **c)** *A. hazarensis* from locality Laram Qilla, Lower Dir, Pakistan (1436 m; locality no. d); d) tadpole of *A. hazarensis* from Margi, Murre, Pakistan (1618 m, locality no. c); **e)** *N. vicina* from Narkanda (2650 m; locality no. 68); **f)** *N. vicina* from Pulga, (2199 m; locality no. 69); **g)** *N. vicina* from Panjpula (2016 m; locality no. 72); **h)** tadpole of *N. vicina* from Panjpula (2016 m; locality no. 72).
