# Supplementary material for: Relict groups of spiny frogs indicate Late Paleogene-Early Neogene trans-Tibet dispersal of thermophile faunal elements
Source: PeerJ. 2021 Jul 15;9:e11793. doi: 10.7717/peerj.11793 (PMC8286701; doi:10.7717/peerj.11793)
Supplement: Supplemental Information 2 [file peerj-09-11793-s002.pdf]

Supplemental Information Fig. S2

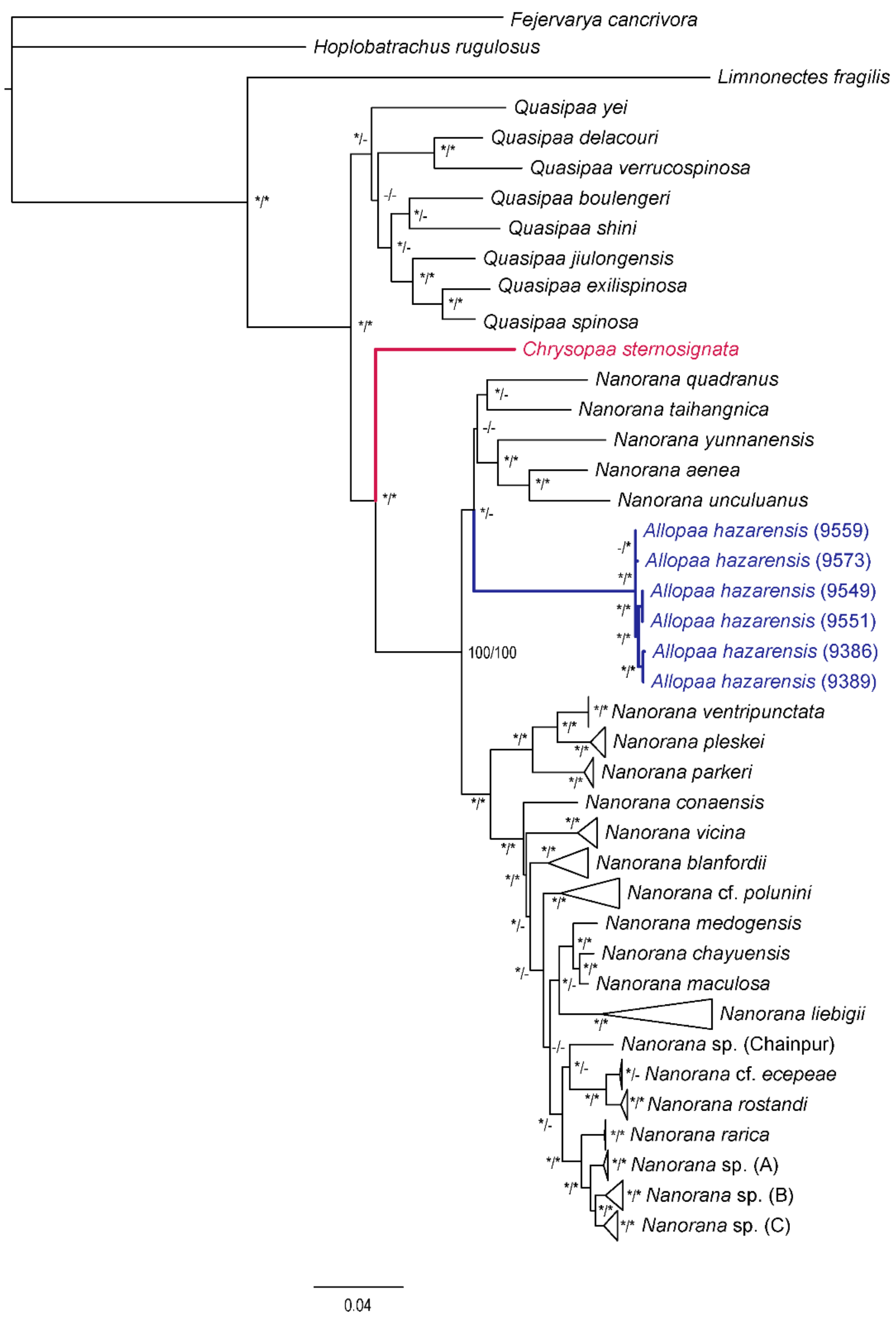

IQ-TREE topology. SH-aLRT (*≥70) and ultrafast bootstrap support (*≥ 90) values are indicated with an asterisk at the respective node.

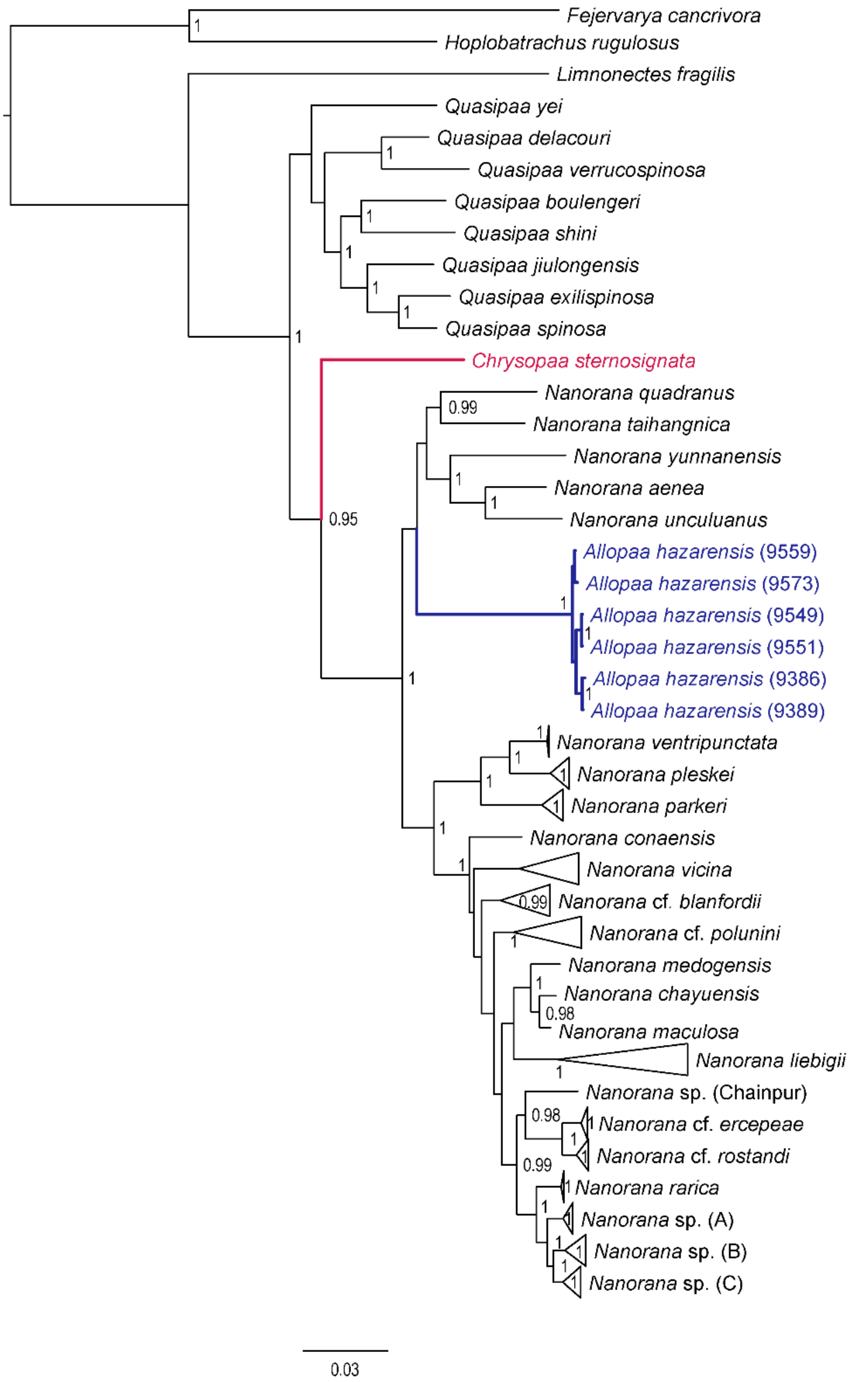

MrBayes tree generated with the standard 4×4 model of DNA substitution. Posterior probabilities ≥ 0.90 are shown at the respective node.
