# Supplementary material for: Relict groups of spiny frogs indicate Late Paleogene-Early Neogene trans-Tibet dispersal of thermophile faunal elements
Source: PeerJ. 2021 Jul 15;9:e11793. doi: 10.7717/peerj.11793 (PMC8286701; doi:10.7717/peerj.11793)
Supplement: Supplemental Information 3 [file peerj-09-11793-s003.pdf]

Supplemental Information Fig. S3

a) **b)**

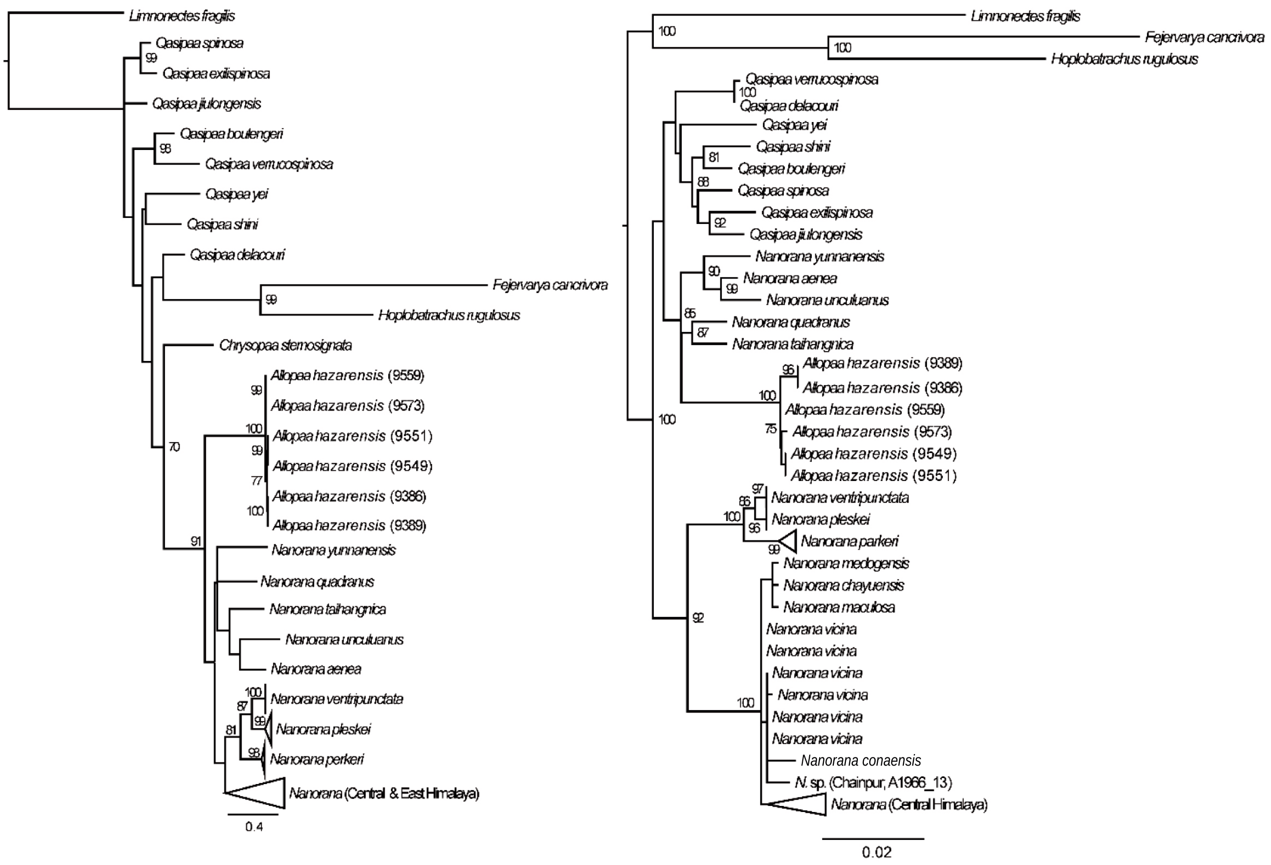

ML tree topology. Trees were inferred with RAxML v.8.2.12 (Stamatakis 2014) based on **a)** mitochondrial 16s+COI, and **b)** nuclear Rag1 sequence data. RAxML was performed with the GTRGAMMA model and 1000 bootstrap replicates; the dataset was partitioned by gene and codon fragments. Numbers at nodes reflect bootstrap values ≥70.

Reference

Stamatakis A (2014) RAxML Version 8: A tool for phylogenetic analysis and post-analysis of large phylogenies. Bioinformatics 30: 1312–1313.
