# Supplementary material for: Relict groups of spiny frogs indicate Late Paleogene-Early Neogene trans-Tibet dispersal of thermophile faunal elements
Source: PeerJ. 2021 Jul 15;9:e11793. doi: 10.7717/peerj.11793 (PMC8286701; doi:10.7717/peerj.11793)
Supplement: Supplemental Information 4 — Locality identifier (Loc) refer to the Fig. 1. Coordinates are given in decimal degrees. §= samples of photographed specimens (see Fig. S1); * = sample localities not shown in Fig. 1 because they lie far to SO China; for reference see map in Hofmann et al. (2019). [file peerj-09-11793-s004.pdf]

Supplemental Information Tab. S1

List of species used in the present study, including sample ID or voucher numbers, sample localities and GenBank accession numbers. Locality identifier (Loc) refers to the Fig. 1. Coordinates are given in decimal degrees. § = samples of photographed specimens (see Fig. S1); * = sample localities not shown in Fig. 1 because they lie far to SO China; for reference see map in Hofmann et al. (2019).

Taxon	SampleID/voucher	Data origin	Loc	N	E	16S	co1	rag1
Allopaa hazarensis	9386	This study	a	34.295	73.258	MW598397	MW603002	MW598465
Allopaa hazarensis	9389	This study	a	34.295	73.258	MW598398	MW603003	MW598466
Allopaa hazarensis	9549	This study	b	33.951	73.468	MW598393	MW603004	MW598461
Allopaa hazarensis	9551	This study	c	33.940	73.465	MW598394	MW603005	MW598462
Allopaa hazarensis	9559	This study	d	34.785	71.986	MW598395	MW603006	MW598463
Allopaa hazarensis	9573	This study	d	34.785	71.986	MW598396	MW603007	MW598464
Chrysopaa sternosignata	USNM:Herp589844	NCBI	e	34.940	69.255	MG700155	MG699938	—
N. aenea		NCBI	3	22.336	103.844	EU979830	KR087830	HM163609
N. cf. *blanfordii*	JS040529_NME	Hofmann et al. 2019	29	27.617	87.233	MN012067	—	MN032491
N. cf. *blanfordii*	JS040531_NME	Hofmann et al. 2019	29	27.617	87.233	MN012068	—	MN032492
N. cf. *blanfordii*	JS040532_NME	Hofmann et al. 2019	29	27.617	87.233	MN012069	—	MN032493
N. cf. *blanfordii*	JS040533_NME	Hofmann et al. 2019	29	27.617	87.233	MN012070	—	MN032494
N. cf. *blanfordii*	JS040534_NME	Hofmann et al. 2019	29	27.617	87.233	MN012071		MN032495
N. cf. *blanfordii*	JS040535_NME	Hofmann et al. 2019	29	27.617	87.233	MN012072	—	MN032496
N. cf. *blanfordii*	JS060520_NME	Hofmann et al. 2019	28	27.173	87.421	MN012073	—	MN032497
N. cf. *blanfordii*	JS060515_NME	Hofmann et al. 2019	27	27.214	87.463	MN012074	—	MN032498
N. cf. *blanfordii*	JS060508_NME	Hofmann et al. 2019	25	27.413	87.734	MN012075	—	MN032499
N. cf. ercepeae	A2016/13_NME	Hofmann et al. 2019	67	29.374	81.137	—	MN012211	—
N. cf. ercepeae	A2017/13_NME	Hofmann et al. 2019	67	29.374	81.137	MN012076	MN012212	MN032500
N. cf. ercepeae	A1_12_NME	Hofmann et al. 2019	63	28.963	82.857	MN012077	MN012213	MN032501
N. cf. ercepeae	A7_12_NME	Hofmann et al. 2019	62	28.855	82.961	MN012078	MN012214	MN032502
N. cf. ercepeae	A4_12_NME	Hofmann et al. 2019	61	28.857	82.976	MN012079	MN012215	MN032503
N. cf. ercepeae	A5_12_NME	Hofmann et al. 2019	61	28.857	82.976	MN012080	MN012216	MN032504
N. cf. ercepeae	A6_12_NME	Hofmann et al. 2019	61	28.857	82.976	MN012081	MN012217	MN032505
N. cf. *polunini*	R15_12_NME	Hofmann et al. 2019	59	28.502	83.129	MN012082	MN012218	MN032506
N. cf. *polunini*	R20_12_NME	Hofmann et al. 2019	57	28.513	83.255	MN012083	—	MN032507
N. cf. *polunini*	SH070507_NME	Hofmann et al. 2019	50	28.060	85.294	MN012084	MN012219	MN032508
N. cf. *polunini*	SH070509_NME	Hofmann et al. 2019	49	28.080	85.295	MN012085	MN012220	MN032509
N. cf. *polunini*	SH070531_NME	Hofmann et al. 2019	46	27.965	85.472	MN012086	MN012221	MN032510
N. cf. *polunini*	R3_09_13_NME	Hofmann et al. 2019	51	28.380	84.065	MN012087	MN012222	MN032511
N. cf. *rarica*	A1961/13_NME	Hofmann et al. 2019	66	29.510	82.090	MN012202	MN012322	—
N. cf. *rarica*	A1970/13_NME	Hofmann et al. 2019	66	29.510	82.090	MN012203	MN012323	MN032606
N. cf. *rarica*	A2015/13_NME	Hofmann et al. 2019	66	29.510	82.090	MN012204	MN012324	MN032607
N. cf. *rarica*	A2019/13_NME	Hofmann et al. 2019	66	29.510	82.090	MN012205	MN012325	MN032608
N. cf. *rarica*	A1965/13_NME	Hofmann et al. 2019	65	29.513	82.092	MN012206	MN012326	MN032609
N. cf. *rarica*	A1960/13_NME	Hofmann et al. 2019	64	29.360	82.200	MN012207	MN012327	—
N. cf. *rostandi*	R1_12_NME	Hofmann et al. 2019	60	28.513	83.033	MN012088	MN012223	MN032512
N. cf. *rostandi*	R2_12_NME	Hofmann et al. 2019	60	28.513	83.033	MN012089	MN012224	MN032513
N. cf. *rostandi*	R3_12_NME	Hofmann et al. 2019	60	28.513	83.033	MN012090	MN012225	MN032514
N. cf. *rostandi*	R4_12_NME	Hofmann et al. 2019	60	28.513	83.033	MN012091	MN012226	MN032515
N. cf. *rostandi*	R11_12_NME	Hofmann et al. 2019	59	28.502	83.129	MN012092	MN012227	MN032516

N. cf. *rostandi*	R12_12_NME	Hofmann et al. 2019	59	28.502	83.129	MN012093	MN012228	MN032517
N. cf. *rostandi*	R13_12_NME	Hofmann et al. 2019	59	28.502	83.129	MN012094	MN012229	MN032518
N. cf. *rostandi*	R14_12_NME	Hofmann et al. 2019	59	28.502	83.129	MN012095	MN012230	MN032519
N. cf. *rostandi*	R16_12_NME	Hofmann et al. 2019	59	28.502	83.129	MN012096	MN012231	MN032520
N. cf. *rostandi*	R6_12_NME	Hofmann et al. 2019	59	28.502	83.129	MN012097	MN012232	MN032521
N. cf. *rostandi*	R7_12_NME	Hofmann et al. 2019	59	28.502	83.129	MN012098	MN012233	MN032522
N. cf. *rostandi*	R8_12_NME	Hofmann et al. 2019	59	28.502	83.129	MN012099	MN012234	MN032523
N. cf. *rostandi*	R9_12_NME	Hofmann et al. 2019	59	28.502	83.129	MN012100	MN012235	MN032524
N. cf. *rostandi*	R17_12_NME	Hofmann et al. 2019	56	28.519	83.264	MN012101	MN012236	MN032525
N. cf. *rostandi*	SH070550_NME	Hofmann et al. 2019	55	28.683	83.591	MN012102	—	MN032526
N. cf. *rostandi*	SH070538_NME	Hofmann et al. 2019	54	28.680	83.594	MN012103	—	MN032527
N. chayuensis	SCUM050410CHX	NCBI	12	25.823	98.858	EU979838	—	HM163587
N. conaensis	KIZ-YP152	NCBI	16	27.991	91.957	EU979834	—	HM163589
N. liebigii	A17_12_NME	Hofmann et al. 2019	56	28.519	83.264	MN012104	MN012237	MN032528
N. liebigii	R18_12_NME	Hofmann et al. 2019	56	28.519	83.264	MN012105	MN012238	MN032529
N. liebigii	SH070515_NME	Hofmann et al. 2019	47	28.099	85.317	MN012106	—	MN032530
N. liebigii	SH0805109_NME	Hofmann et al. 2019	45	27.673	86.240	MN012107	—	MN032531
N. liebigii	SH080506_NME	Hofmann et al. 2019	42	27.609	86.295	MN012108	—	MN032532
N. liebigii	SH080554_NME	Hofmann et al. 2019	41	27.718	86.311	MN012109	—	MN032533
N. liebigii	SH080536_NME	Hofmann et al. 2019	38	27.691	86.343	MN012110	MN012239	—
N. liebigii	SH080537_NME	Hofmann et al. 2019	38	27.691	86.343	MN012111	MN012240	MN032534
N. liebigii	SH080538_NME	Hofmann et al. 2019	38	27.691	86.343	MN012112	—	MN032535
N. liebigii	SH080524_NME	Hofmann et al. 2019	37	27.694	86.351	MN012113	MN012241	MN032536
N. liebigii	SH080534_NME	Hofmann et al. 2019	37	27.694	86.351	MN012114	—	MN032537
N. liebigii	Ne16_13_NME	Hofmann et al. 2019	36	27.584	86.411	MN012115	MN012242	MN032538
N. liebigii	Ne17_13_NME	Hofmann et al. 2019	36	27.584	86.411	MN012116	MN012243	MN032539
N. liebigii	Ne12_13_NME	Hofmann et al. 2019	34	27.584	86.594	MN012117	MN012244	MN032540
N. liebigii	Ne10_13_NME	Hofmann et al. 2019	33	27.586	86.635	MN012118	MN012245	MN032541
N. liebigii	JS040512_NME	Hofmann et al. 2019	30	27.631	87.224	MN012119	MN012246	MN032542
N. liebigii	JS040513_NME	Hofmann et al. 2019	30	27.631	87.224	MN012120	MN012247	MN032543
N. liebigii	JS060518_NME	Hofmann et al. 2019	28	27.173	87.421	MN012121	—	MN032544
N. liebigii	JS060511_NME	Hofmann et al. 2019	26	27.296	87.535	MN012122	—	MN032545
N. liebigii	JS060509_NME	Hofmann et al. 2019	25	27.413	87.734	MN012123	—	MN032546
N. liebigii	JS060502_NME	Hofmann et al. 2019	24	27.407	87.752	—	—	MN032547
N. liebigii	JS060503_NME	Hofmann et al. 2019	24	27.407	87.752	MN012124	—	MN032548
N. liebigii	KIZ-RDXZL1	NCBI	23	27.485	88.907	DQ118499	KJ810987	HM163607
N. maculosa	YNU-HU2002308	NCBI	8	24.400	100.800	EU979835	—	HM163588
N. medogensis	SYNU-XZ35	NCBI	13	29.367	95.583	DQ118506	—	HM163590
N. parkeri	N6_06_NME	Hofmann et al. 2019	22	29.589	90.214	MN012125	MN012248	—
N. parkeri	N7_06_NME	Hofmann et al. 2019	22	29.589	90.214	MN012126	MN012249	MN032549
N. parkeri	N8_06_NME	Hofmann et al. 2019	22	29.589	90.214	MN012127	MN012250	MN032550
N. parkeri	N5_06_NME	Hofmann et al. 2019	21	29.573	90.433	MN012128	MN012251	—
N. parkeri	TP10_06_NME	Hofmann et al. 2019	21	29.573	90.433	MN012129	MN012252	—
N. parkeri	TP11_06_NME	Hofmann et al. 2019	21	29.573	90.433	MN012130	MN012253	—
N. parkeri	TP8_06_NME	Hofmann et al. 2019	21	29.573	90.433	—	MN012254	—
N. parkeri	TP9_06_NME	Hofmann et al. 2019	21	29.573	90.433	MN012131	MN012255	—
N. parkeri	N10_06_NME	Hofmann et al. 2019	20	29.578	90.435	MN012132	MN012256	—
N. parkeri	N9_06_NME	Hofmann et al. 2019	20	29.578	90.435	MN012133	MN012257	MN032551
N. parkeri	TP1_06_NME	Hofmann et al. 2019	20	29.578	90.435	MN012134	MN012258	MN032552
N. parkeri	TP2_06_NME	Hofmann et al. 2019	20	29.578	90.435	MN012135	—	MN032553

N. parkeri	TP3_06_NME	Hofmann et al. 2019	20	29.578	90.435	MN012136	MN012259	—
N. parkeri	CAS801L	Hofmann et al. 2019	19	30.090	90.480	MN012137	MN012260	MN032554
N. parkeri	CAS802L	Hofmann et al. 2019	19	30.090	90.480	MN012138	MN012261	MN032555
N. parkeri	CAS803L	Hofmann et al. 2019	19	30.090	90.480	MN012139	MN012262	—
N. parkeri	CAS804L	Hofmann et al. 2019	19	30.090	90.480	MN012140	MN012263	—
N. parkeri	CAS805L	Hofmann et al. 2019	19	30.090	90.480	MN012141	MN012264	—
N. parkeri	A6AL_NME	Hofmann et al. 2019	18	30.156	90.647	MN012142	—	—
N. parkeri	JS0507B01_NME	Hofmann et al. 2019	17	30.378	90.908	MN012143	MN012265	MN032556
N. parkeri	JS0507B02_NME	Hofmann et al. 2019	17	30.378	90.908	MN012144	MN012266	MN032557
N. parkeri	JS0507B03_NME	Hofmann et al. 2019	17	30.378	90.908	MN012145	MN012267	MN032558
N. parkeri	JS0507B04_NME	Hofmann et al. 2019	17	30.378	90.908	MN012146	MN012268	MN032559
N. parkeri	JS0507B05_NME	Hofmann et al. 2019	17	30.378	90.908	MN012147	MN012269	MN032560
N. parkeri		NCBI	17	30.378	90.908	KP317482	KP317482	HM163584
N. parkeri	N1_06_NME	Hofmann et al. 2019	15	31.166	92.061	MN012148	MN012270	—
N. parkeri	N2_06_NME	Hofmann et al. 2019	15	31.166	92.061	MN012149	MN012271	—
N. parkeri	N3_06_NME	Hofmann et al. 2019	15	31.166	92.061	MN012150	MN012272	MN032561
N. parkeri	N4_06_NME	Hofmann et al. 2019	15	31.166	92.061	MN012151	MN012273	—
N. parkeri	TP4_06_NME	Hofmann et al. 2019	15	31.166	92.061	MN012152	MN012274	—
N. parkeri	TP5_06_NME	Hofmann et al. 2019	15	31.166	92.061	MN012153	MN012275	—
N. parkeri	TP6_06_NME	Hofmann et al. 2019	15	31.166	92.061	MN012154	MN012276	—
N. parkeri	TP7_06_NME	Hofmann et al. 2019	15	31.166	92.061	MN012155	MN012277	—
N. parkeri	CIB-XM1096	NCBI	14	29.649	94.361	DQ118498	KJ811345	—
N. pleskei	KQ47_14_NME	Hofmann et al. 2019	6	30.216	101.500	MN012156	MN012278	MN032562
N. pleskei	KQ1_14_NME	Hofmann et al. 2019	5	30.377	101.675	MN012157	MN012279	MN032563
N. pleskei	KQ10_14_NME	Hofmann et al. 2019	5	30.377	101.675	MN012158	MN012280	MN032564
N. pleskei	KQ11_14_NME	Hofmann et al. 2019	5	30.377	101.675	MN012159	MN012281	MN032565
N. pleskei	KQ13_14_NME	Hofmann et al. 2019	5	30.377	101.675	MN012160	MN012282	MN032566
N. pleskei	KQ15_14_NME	Hofmann et al. 2019	5	30.377	101.675	MN012161	MN012283	—
N. pleskei	KQ17_14_NME	Hofmann et al. 2019	5	30.377	101.675	MN012162	MN012284	MN032567
N. pleskei	KQ18_14_NME	Hofmann et al. 2019	5	30.377	101.675	MN012163	MN012285	MN032568
N. pleskei	KQ19_14_NME	Hofmann et al. 2019	5	30.377	101.675	MN012164	MN012286	—
N. pleskei	KQ20_14_NME	Hofmann et al. 2019	5	30.377	101.675	MN012165	MN012287	MN032569
N. pleskei	KQ9_14_NME	Hofmann et al. 2019	5	30.377	101.675	MN012166	MN012288	—
N. pleskei	CAS201	Hofmann et al. 2019	4	33.467	102.750	MN012167	MN012289	MN032570
N. pleskei	CAS202	Hofmann et al. 2019	4	33.467	102.750	MN012168	MN012290	MN032571
N. pleskei		NCBI	4	33.467	102.750	HQ324232	HQ324232	HM163586
N. quadranus	SCUM20045195CJ	NCBI	2	31.683	103.850	DQ118514	—	HM163591
Nanorana sp. [A]	R5_12_NME	Hofmann et al. 2019	60	28.513	83.033	MN012169	MN012291	MN032572
Nanorana sp. [A]	R10_12_NME	Hofmann et al. 2019	59	28.502	83.129	MN012170	—	MN032573
Nanorana sp. [A]	KQ2_12_NME	Hofmann et al. 2019	58	28.501	83.198	MN012171	MN012292	MN032574
Nanorana sp. [A]	SH070556_NME	Hofmann et al. 2019	53	28.622	83.662	MN012172	MN012293	MN032575
Nanorana sp. [A]	A1963/13_NME	Hofmann et al. 2019	52	28.400	83.700	MN012173	MN012294	—
Nanorana sp. [B]	R1_09_13_NME	Hofmann et al. 2019	51	28.380	84.065	MN012174	MN012295	MN032576
Nanorana sp. [B]	R2_09_13_NME	Hofmann et al. 2019	51	28.380	84.065	MN012175	MN012296	MN032577
Nanorana sp. [B]	R4_09_13_NME	Hofmann et al. 2019	51	28.380	84.065	MN012176	MN012297	MN032578
Nanorana sp. [B]	SH070510_NME	Hofmann et al. 2019	48	28.074	85.302	MN012177	—	MN032579
Nanorana sp. [C]	SH080591_NME	Hofmann et al. 2019	44	27.686	86.252	MN012178	MN012298	MN032580
Nanorana sp. [C]	SH080592_NME	Hofmann et al. 2019	44	27.686	86.252	MN012179	MN012299	MN032581
Nanorana sp. [C]	SH080593_NME	Hofmann et al. 2019	44	27.686	86.252	MN012180	MN012300	MN032582
Nanorana sp. [C]	SH080594_NME	Hofmann et al. 2019	44	27.686	86.252	MN012181	MN012301	MN032583

Nanorana sp. [C]	SH080570_NME	Hofmann et al. 2019	43	27.697	86.275	MN012182	MN012302	MN032584
Nanorana sp. [C]	SH080571_NME	Hofmann et al. 2019	43	27.697	86.275	MN012183	MN012303	MN032585
Nanorana sp. [C]	SH080572_NME	Hofmann et al. 2019	43	27.697	86.275	MN012184	MN012304	MN032586
Nanorana sp. [C]	SH080553_NME	Hofmann et al. 2019	41	27.718	86.311	MN012185	MN012305	MN032587
Nanorana sp. [C]	SH080555_NME	Hofmann et al. 2019	41	27.718	86.311	MN012186	MN012306	MN032588
Nanorana sp. [C]	SH080545_NME	Hofmann et al. 2019	40	27.703	86.337	MN012187	MN012307	MN032589
Nanorana sp. [C]	SH080546_NME	Hofmann et al. 2019	40	27.703	86.337	MN012188	MN012308	MN032590
Nanorana sp. [C]	SH080548_NME	Hofmann et al. 2019	40	27.703	86.337	MN012189	MN012309	MN032591
Nanorana sp. [C]	SH080551_NME	Hofmann et al. 2019	40	27.703	86.337	MN012190	MN012310	MN032592
Nanorana sp. [C]	SH080552_NME	Hofmann et al. 2019	40	27.703	86.337	MN012191	MN012311	—
Nanorana sp. [C]	SH080512_NME	Hofmann et al. 2019	39	27.595	86.340	MN012192	MN012312	MN032593
Nanorana sp. [C]	SH080523_NME	Hofmann et al. 2019	37	27.694	86.351	MN012193	MN012313	MN032594
Nanorana sp. [C]	Ne13_13_NME	Hofmann et al. 2019	35	27.576	86.514	MN012194	MN012314	MN032595
Nanorana sp. [C]	Ne1_13_NME	Hofmann et al. 2019	32	27.689	86.731	MN012195	MN012315	MN032596
Nanorana sp. [C]	Ne2_13_NME	Hofmann et al. 2019	32	27.689	86.731	MN012196	MN012316	MN032597
Nanorana sp. [C]	Ne9_13_NME	Hofmann et al. 2019	31	27.671	86.765	MN012197	MN012317	MN032598
Nanorana sp. [Chainpur]	A1966/13_NME	Hofmann et al. 2019	67	29.374	81.137	MN012198	MN012318	MN032599
N. vicina	2Bhan_RAS	Hofmann et al. 2019	74	32.873	75.858	MN012199	MN012319	—
N. vicina	1G_RAS	Hofmann et al. 2019	73	32.777	75.947	—	MN012320	MN032600
N. vicina	1Pa_RAS [§]	Hofmann et al. 2019	72	32.528	75.991	—	MN012321	MN032601
N. vicina	2Ba_RAS	Hofmann et al. 2019	71	31.783	77.068	—	—	MN032602
N. vicina	2Baj_RAS	Hofmann et al. 2019	70	31.821	77.112	—	—	MN032603
N. vicina	2Pul_RAS [§]	Hofmann et al. 2019	69	31.996	77.448	MN012200	—	MN032604
N. vicina	782_RAS [§]	Hofmann et al. 2019	68	31.261	77.450	MN012201	—	MN032605
N. taihangnica		NCBI	1	35.265	112.090	KF199146	KF199146	HM163608
N. unculuanus	YNUHU2002502601	NCBI	7	24.447	100.834	DQ118491	—	HM163595
N. ventripunctata	SCUM045887WD	NCBI	11	27.830	99.701	EU979839	KJ810985	HM163585
N. ventripunctata	SH050538_NME	Hofmann et al. 2019	10	27.788	99.855	MN012208	MN012328	MN032610
N. ventripunctata	SH050539_NME	Hofmann et al. 2019	10	27.788	99.855	MN012209	MN012329	MN032611
N. yunnanensis		NCBI	9	27.724	100.789	KF199150	KF199150	HM163593
Q. boulengeri	YNU-HU20025106	NCBI	80	28.811	105.831	KX645665	KX645665	HM163604
Q. delacouri	FMNH255623	NCBI	82	19.018	104.799	EU979810	EU979664	HM163600
Q. exilispinosa	KF199151	NCBI	81*	22.396	114.109	KF199151	KF199151	HM163610
Q. jiulongensis	KF199149	NCBI	75*	27.750	117.683	KF199149	KF199149	HM163603
Q. shini	KF199148	NCBI	76	25.598	109.935	KF199148	KF199148	HM163602
Q. spinosa		NCBI	77	24.481	99.047	NC_013270	NC_013270	HM163606
Q. verrucospinosa		NCBI	78	21.789	101.142	KF199147	KF199147	HM163599
Q. yei	HNNU0908I061	NCBI	79*	31.798	115.407	KJ842105	KJ842105	HM163596
Fejervarya cancrivora		NCBI				EU652694	EU652694	HM163581
Hoplobatrachus rugulosus		NCBI				NC_019615	NC_019615	HM163612
Limnonectes fragilis	ZNAC11006	NCBI				AY899241	AY899241	HM163611

Reference

Hofmann, S. et al. Phylogeny of spiny frogs *Nanorana* (Anura: Dicroglossidae) supports a Tibetan origin of a Himalayan species group. Ecol Evol 9, 14498-14511, doi:10.1002/ece3.5909 (2019).
